# Bayesian dynamic profiling and optimization of important ranked energy from gray level co-occurrence (GLCM) features for empirical analysis of brain MRI

**DOI:** 10.1038/s41598-022-19563-0

**Published:** 2022-09-13

**Authors:** Lal Hussain, Areej A. Malibari, Jaber S. Alzahrani, Mohamed Alamgeer, Marwa Obayya, Fahd N. Al-Wesabi, Heba Mohsen, Manar Ahmed Hamza

**Affiliations:** 1 Department of Computer Science and Information Technology, King Abdullah Campus Chatter Kalas, University of Azad Jammu and Kashmir, Muzaffarabad, 13100 Azad Kashmir Pakistan; 2 Department of Computer Science and Information Technology, Neelum Campus, University of Azad Jammu and Kashmir, Athmuqam, 13230 Azad Kashmir Pakistan; 3 Department of Industrial and Systems Engineering, College of Engineering, Princess Nourah Bint Abdulrahman University, P.O. Box 84428, Riyadh, 11671 Saudi Arabia; 4 Department of Industrial Engineering, College of Engineering at Alqunfudah, Umm Al-Qura University, Mecca, Saudi Arabia; 5 Department of Information Systems, College of Science and Art at Mahayil, King Khalid University, Abha, Saudi Arabia; 6 Department of Biomedical Engineering, College of Engineering, Princess Nourah Bint Abdulrahman University, P.O. Box 84428, Riyadh, 11671 Saudi Arabia; 7 Department of Computer Science, College of Science and Art at Mahayil, King Khalid University, Abha, Saudi Arabia; 8 Department of Computer Science, Faculty of Computers and Information Technology, Future University in Egypt, New Cairo, 11835 Egypt; 9 Department of Computer and Self Development, Preparatory Year Deanship, Prince Sattam Bin Abdulaziz University, Al-Kharj, Saudi Arabia

**Keywords:** Cancer, Health care

## Abstract

Accurate classification of brain tumor subtypes is important for prognosis and treatment. Researchers are developing tools based on static and dynamic feature extraction and applying machine learning and deep learning. However, static feature requires further analysis to compute the relevance, strength, and types of association. Recently Bayesian inference approach gains attraction for deeper analysis of static (hand-crafted) features to unfold hidden dynamics and relationships among features. We computed the gray level co-occurrence (GLCM) features from brain tumor meningioma and pituitary MRIs and then ranked based on entropy methods. The highly ranked Energy feature was chosen as our target variable for further empirical analysis of dynamic profiling and optimization to unfold the nonlinear intrinsic dynamics of GLCM features extracted from brain MRIs. The proposed method further unfolds the dynamics and to detailed analysis of computed features based on GLCM features for better understanding of the hidden dynamics for proper diagnosis and prognosis of tumor types leading to brain stroke.

## Introduction

For proper treatment, prognosis, planning and monitoring of brain tumor, an accurate intelligent algorithm is required^1^. The brain tumor types is still a challenges, as MRIs cannot make definitive diagnosis of brain tumor subtypes^2^. Various computer assisted and machine learning algorithms have been developed to assist the doctors for proper diagnosis of brain tumor subtypes. Feature extraction is one of the most crucial parts, which require most relevant methods to generate the features from raw images. Selecting the most relevant features is tedious task which require the knowledge domain. Researchers in the recent past computed the different categories of features including texture, morphological, scale invariant Fourier transform (SIFT), elliptic Fourier descriptors (EFDs) for prediction of pathologies in medical imaging problems^3–8^. These features are utilized as input to different machine learning algorithms^9^. Researchers developed different machine learning classification algorithms for brain tumor type classification^10–24^.

Accurate classification of brain tumor subtypes is important for treatment, planning, prognosis, and monitoring etc.^1^. MRI^25^ provides valuable diagnostic information to characterize brain tumors^2^, but challenges lie in the ability to classify different tumor types. In contrast to pathology, MRIs cannot make definitive diagnoses of brain tumor subtypes. The machine learning algorithms such as support vector machines (SVM)^10–19^, Adaboost^21^, random forest^20^ and instance-based K-Nearest using log^19,22^ relied on hand-crafted features including discrete wavelet transform (DWT)^10,11,16,20,23,24^, gray-level co-occurrence matrix (GLCM)^10,11,14,17^ and genetic algorithm^26^ etc.

The previous studies include the multiclass classification based on machine learning and deep learning using diver feature extraction approaches. However, a more comprehensive analysis to determine the associations and other Bayesian measures can further strengthen our analysis to unfold the hidden dynamics for further improving the diagnostic capabilities. The parametric information from the data in the recent studies have been investigated using a probabilistic propagation algorithm (Bayes Rule) by applying Bayesian networks (BNs). The associations and degree of uncertainty of the variables varies from different sources such as numerical data, empirical data, expert opinion etc. to capture the conditional dependencies of a variable upon others^27^. BNs have successfully been utilized in many studies by different researchers such as Kocian et al.^28^, Amaral et al.^29^, Laurila-Pant et al.^30^, Zhang et al.^31^. The causal relationships can be studied between variables which compute the probabilities of a variable when other variables in the model are known. Moreover, the Monte Carlo analysis (MCA) can be used at random sampling of probability distribution functions (PDF) to denote the inputs of Bayesian model to produce hundreds or thousands of possible outcomes^32^. Recently, BNs have successfully been utilized in many applications ranging from predicting energy crop yield^33^, prediction of coffee rust disease using Bayesian networks^34^, sustainable planning and management decision^35^, etc. The Bayesian networks computed the interrelation among variables that impacts climate changes scenarios in agriculture^36^. Moreover, recently, Lu et al.^37^ utilized BNs to investigate the complex causal interactions between environments and plant diseases. Hussain et al.^38^ computed the morphological features and determine the association from prostate cancer MRIs.

In the past, researchers utilized machine learning and deep learning algorithms to classify the brain tumor types and other cancerous pathologies^5,39–42^. The machine learning algorithms requires hand-crafted features for training the models and predicting for new examples. The classification performance merely based on type and relevancy of extracted feature. The researchers utilized the different features extracting approaches including texture, morphological, geometric, scale invariant feature transform (SIFT), elliptic Fourier descriptors (EFDs) etc. as extracting the most relevant feature is challenging which can be very helpful to improve the classification performance. The classification tasks based on computed features can only provide the classification performance, however association among features, in-depth parent–child relationships, strength of relationship, degree and features incoming and outgoing force, computed segments profile analysis, impact of posterior probabilities can further unfold the hidden and nonlinear dynamics of extracted features, which can be very much helpful for the concerned radiologists and health practitioners for making the wise decisions for further prognosis and diagnosis of brain tumor. The aim of this study was to apply the Bayesian inference approach for comprehensive analysis in order to unfold the nonlinear and hidden dynamics present in brain tumor MRI types (meningioma and pituitary) by extracting the gray-level co-occurrence (GLCM) features and to compute the associations, and strength of relationships among features. The Bayesian approach recently gain its popularity and utilized in many biomedical signal and image processing problems. The Bayesian inference estimates the posterior which can be produced from a weighted combination of local estimates also known as likelihood and estimates in surrounding spatial units. Researchers are developing intelligent methods based on Machine learning which requires the extraction of most relevant features. Our research objective was multifold, as the Bayesian analysis is based on target node for which we first requires the top ranked features among the extracted features. We first we computed the GLCM based texture features from brain tumor meningioma and pituitary images. We then ranked the features based on entropy value using MATLAB diagnosticFeatureDesigner tool. The higher the entropy value indicate the more important feature. Secondly, the high ranked feature was selected as our target node and then we further computed the detailed Bayesian analysis with other features to compute the underlying hidden dynamics among the nodes. We computed the relationship analysis among the extracted nodes using mutual information (MI), Kullback–Leibler (KL) divergence and Pearson’s correlation. The strength of relationship was computed using arc analysis with 3D mapping. We then computed the parent–child relationship and nodes force between the nodes. The association graph for segment profile analysis was computed for further analysis. Moreover, the network performance and significance of prominance was computed using tarnando diagram. The radar chart also reflects the importance of significance of target node with other nodes at different selected states. In most of the states, we obtained significant results using the statistical tests. Which indicates the stronger binding of target Energy node with other computed GLCM features. The target’s posterior probabilities at selected states also shows the great influence on target variable. The network performance also shows the highest significant results with respect to reliability, occurrence, precision, and Gini index. This study can be very helpful to understand the deeper insights to further investigate the hidden dynamics in the MRI signals from Brain tumor types and can play a vital role for providing improved diagnostic system. The concerned radiologists can utilize this analysis as a biomarker for improved diagnosis and prognosis, treatment planning, recurrence.

## Materials and methods

### Dataset

A publicly dataset was taken available at (https://github.com/chengjun583/brainTumorRetrieval) which is described in^43,44^. It comprised of 3064 T1-weighted contrast enhanced images from 233 patients. The dataset contains three types of brain tumor such as and meningiomas (708 slices), pituitary tumor (930 slices) and gliomas (1426 slices). In this study, we applied the Bayesian inference approach for comprehensive analysis between the two selected classes of pituitary and meningioma.

### Feature extraction

In machine learning, the first and foremost step is to compute the most relevant features which are quantitative values computed on images. Researchers proposed a set hybrid and single features for classifying images^5^. Likewise, different image segmentation and classification algorithms are then utilized to classify malignant or benign cases^45^.

Feature plays a vital role in image processing. After applying image processing techniques to the captured image, different feature extracting techniques are applied to obtain the features used in classification. The behavior of an image can be defined by its features. Feature extraction is a type of dimensionality reduction in image processing. Extracting most relevant and required information from the data is one of the main objectives of feature extraction^46^.

In previous studies, numerous researchers have extracted many features for detecting various imaging pathologies by considering texture, shape-based morphologies, and image scaling and rotation changes and complex dynamics using SIFT, morphological, textural, EFDs and some other most relevant features regarding the nature of the problem of interest^4,5,7,47,48^. The feature extracted developed and employed in our previous studies are detailed in^7,49–53^. In this study, we first computed the Gray-level co-occurrence matrix-based texture features.

### Gray-level co-occurrence matrix (GLCM)

The GLCM based texture features extracted from input images by performing transition on two pixels with gray level. GLCM features are originally proposed in 1973^54^ which characterizes the texture properties by utilizing diverse quantities yielded from 2nd order statistics. Two steps are used to compute GLCM features. Firstly, the pair-wise spatial co-occurrences of image pixels are separated by a distance d in a particular direction angle θ. A spatial relationship is created between two pixels i.e. the neighboring and reference pixels. Secondly, scaler quantities are computed to characterize several aspects of an image by forming gray level co-occurrence matrix which contain several gray level pixel combinations of different values of an image^54^. The GLCM is a square matrix of order M × M, where M denote the gray level number of image. The distance d = 1, 2, 3, 4 and angle 0°, 45°, 90° and 135° direction are used to obtain GLCM features. The GLCM contain an element $P(i,\;j,\;d,\;\theta )$, which shows two pixels probability separated by a distance d and angle θ having gray levels of i and j^55–57^. The detailed mathematical formulations are described and utilized in^5,58–60^. We extracted the GLCM texture features from Brain tumor types developed in MATLAB and utilized in many recent renowned studies for texture analysis^61–64^ available at https://www.mathworks.com/matlabcentral/fileexchange/22187-glcm-texture-features.

### Feature importance

After computing the features from images, all features are not contributing equally, as few features contribute less and few other more. Their importance can be computed using the feature ranking algorithms. The feature ranking algorithm is used to rank the importance of features^65^. We used the feature importance ranking (FIR) algorithms developed in MATLAB^66^ available at https://www.mathworks.com/help/predmaint/ref/diagnosticfeaturedesigner-app.html.

The importance of the extracted features was computed based on the entropy values. Entropy is used in many applications of medical systems to compute their nonlinear dynamical measures present in these systems. Yu et al.^66^ developed MATLAB tool with a total of 30 FIR methods integrated that utilized the feature selection and intelligent diagnosis in real world application. All the ranking methods are detailed in our recent study^67^. For the current study, we utilized the method (17) fir_mat_entropy, which computes the features based on relative entropy also called as Kullback–Leibler distance^68^. The entropy is a measure of randomness which computes the nonlinear dynamics as detailed in^49,69,70^. The higher the entropy values indicate the more complex systems with interacting components and accordingly is the more important feature. So, among the extracted GLCM features, the Energy feature with higher entropy (3.0693) was yielded as our high ranked feature. We then chosen Energy as our target node, and Bayesian analysis was applied with the top ranked feature to further explore the associations, and relationships with other features. Multiple interacting modules of biological systems produce biological signals. which show different arrangements in a complex rhythm. Due to structural part malfunctions and decreased interactions in coupling functions, these rhythms and patterns are disrupted. After ranking the features, the top ranked feature was Energy with higher entropy value. We then kept this feature as our target variable and applied the Bayesian inference approach for further comprehensive analysis with other features so we can develop multiple interacting relationships with the top ranked feature, which could be used as a biomarker for further enhanced diagnosis and prognosis of brain tumor.

### Bayesian network analysis

The causal effect and their relationship was computed using Bayesian inference approach using directed acyclic graph (DAG)^71^. The Bayesian networks compute the conditional joint probabilities to determine the dependencies between the attributes. This is a probabilistic graphical network and is represented by a directed acyclic graph of nodes denoting the variables and arcs denote dependence relationships among the variables. Bayesian networks denote the joint probability distribution (JPD) over all variables represented by nodes in the graph. If $x_{i}$ denote some value of variable $X_{i}$ and $Pa_{i}$ represent some set of values for parents of $X_{i}$, then $P(X_{i} { }|Pa_{i} )$ represent conditional probability distribution^33^. The joint probability distribution P of a Bayesian network B = (G, P) can mathematically expressed as:

1 $$ P\left( {X_{1} ,\;X_{2} , \ldots ,\;X_{n} } \right) = \mathop \prod \limits_{i = 1}^{n} P\left( {X_{i} {|}Pa \left( {X_{i} } \right)} \right) $$

Here $Pa \left( {X_{i} } \right)$ denotes the set of random variables associated with the parents of the nodes corresponding to variable $X_{i}$.

The posterior probability is thus computed by utilizing this algorithm through inference of variable of interest. We used BayesiaLab V10 for further analysis^72^ utilizing the supervised learning algorithms to search optimal model.

#### Mutual information (MI)

To compute MI, first Shannon entropy^73^ was computed:

2 $$ H\left( X \right) = - \mathop \sum \limits_{x \in X} p\left( X \right)log_{2} p\left( X \right) $$

The difference between the marginal entropy of target variable and conditional entropy of predicted variable was computed using MI^73^, mathematically:

3 $$ MI\left( {X,Y} \right) = H\left( X \right) - H (X|Y) $$

Which is equivalent to:

4 $$ MI\left( {X,Y} \right) = \mathop \sum \limits_{x \in X} \mathop \sum \limits_{y \in X} p\left( {X,Y} \right){ }log_{2} \frac{{p\left( {X,Y} \right)}}{p\left( X \right)p\left( Y \right)} $$

Moreover, conditional Mutual Information (CMI) is defined as:

5 $$ CMI\left( {X,Y|Z} \right) = \mathop \sum \limits_{x \in X} \mathop \sum \limits_{y \in X} \mathop \sum \limits_{y|z \in X} p\left( {X,Y|Z} \right){ }log_{2} \frac{p(X,Y|Z)}{{p\left( {X|Z} \right)p(Y|Z)}} $$

The joint probability distribution (JPD) of variable X and Y is denoted by p (X, Y). Whereas p(X) and p(Y) represent marginal distribution of X and Y respectively. The relevant Gaussian distribution of co-variance matrix variables X_1_, X_2_, X_3_, …. X_n_^74^ computed as:

$$ H\left( X \right) = \log \left( {2\pi e} \right)^{\frac{n}{2}} \left| C \right|^{{\frac{ - 1}{2}}} $$

The MI and CMI2 can be computed using following mathematical transformation function:

$$ MI\left( {X,Y} \right) = \frac{1}{2}\log \frac{{\left| {C\left( X \right)\left| \times \right|C\left( X \right)} \right|}}{{\left| {C\left( {X,Y} \right)} \right|}} $$

To correct under estimation of CM1^75^, the CMI2 is used to integrate the interventional probability.

### Statistical analysis

We computed the GLCM features from pituitary and meningioma MRIs using MATLAB. We then provided the feature matrix to BayesiaLab for further detailed analysis. We conducted the analysis using BayesiaLab 7.0. We used the BayesiaLab with minimum description length of candidate network in its score-based algorithm to compare the Bayesian network structure^76^. The statistical independence test (GKL-test; p-values > 0.05) was used to validate the connections among the descriptors which were identified by the learning algorithm. The p-values or independence probabilities were utilized to check the significance of each individual relationship between the nodes or between the nodes and the target node^77,78^.

### Exploratory analysis of the unsupervised network

The exploratory analysis can be utilized to determine the potential relationship between variables of interest^79^. We can further explore the global analysis of problem of interest by computing influence between nodes and influence of nodes under investigation. We build our model by learning unsupervised learning algorithm utilizing maximum spanning tree algorithm approach developed in BayesiaLab V10^80^. This method reduces the search space efficiently to a partially directed acyclic graph (PD AG) smaller than space of Bayesian networks (DAGs) represent equivalent classes evaluated during each search by computing directly their scores. We also computed maximum spanning tree (MWST). A lowest minimum description length (MDL) value shows the best trade-off between complexity and data representation.

### Sensitivity analysis

A detailed sensitivity analysis was performed to check the relationship among the nodes in the selected network. To understand the relationship between the nodes, we computed the highest and lowest values of Pearson’s correlation, mutual information, Kullback–Leibler divergence and node force between the nodes was examined globally on the network. The mutual information examined the probabilistic dependencies between the nodes in the network. The Pearson’s correlation computes linear strength of the relationship between the nodes, whereas the Kullback–Leibler divergence was utilized to measure information gain from assuming a joint relationship between two variables in the network compared to an assumption of independence. The node force was also computed, the highest node force indicates that there is more direct relationship and greater dependence with other nodes. The sensitivity of each node was determined in tornado plots that display the influence of knowledge of each node value on the probability of each descriptor and provides information on the maximum strength of the individual relationships between each node and descriptor. The lowest and highest probability values are displayed from the tornado plots to achieve the tornado plots for each node from hard evidence placed on the corresponding descriptor state.

In a Bayesian network (BN), the sensitivity analysis is conducted to determine the most critical factors for a specific result of a specific scenario. This analysis provides the strengths or magnitude of the two-way association between the child and parent node. Using sensitivity analysis, we analyse the impact on other parameters or nodes. Two types are variations are considered, simple either variations are made in only one parameter, or complex in which multiple parameters are considered. The joint probability distribution and network parameters are used for reliable, authentic, and holistic^81,82^. The BNs are considered to exhibit a practical and robust interaction between the considered variables through the induced variations in the selected parameters. In this study, the sensitivity analysis was performed by conducting BayesiaLab package called “Tornado Charts”. This chart displays the minimum and maximum contribution of all the variables in a model towards a specific node and state which is specified as the target node and state. The confidence and consistency level of the sensitivity analysis using the BN model are verified by validating the model^83–85^ to verify different conditions.

### Segment profile analysis of energy

The analysis was also done using segment profile analysis using Radar chart for normalized mean values conditionally to energy for all other GLCM features. The significance was tested using Bayesian test (Best) and NHST *t* test (a frequentist test). Using NHST *t* test, the two tailed *t* test is utilized for null hypothesis significance testing. The Bayesian (Best test) is detailed by Kruschke^86^ which follow the student’s *t* distribution. Moreover, 95% confidence interval (CI) is utilized. When the mean values are estimated significant, a square is added next to the Label.

**Figure 1:**
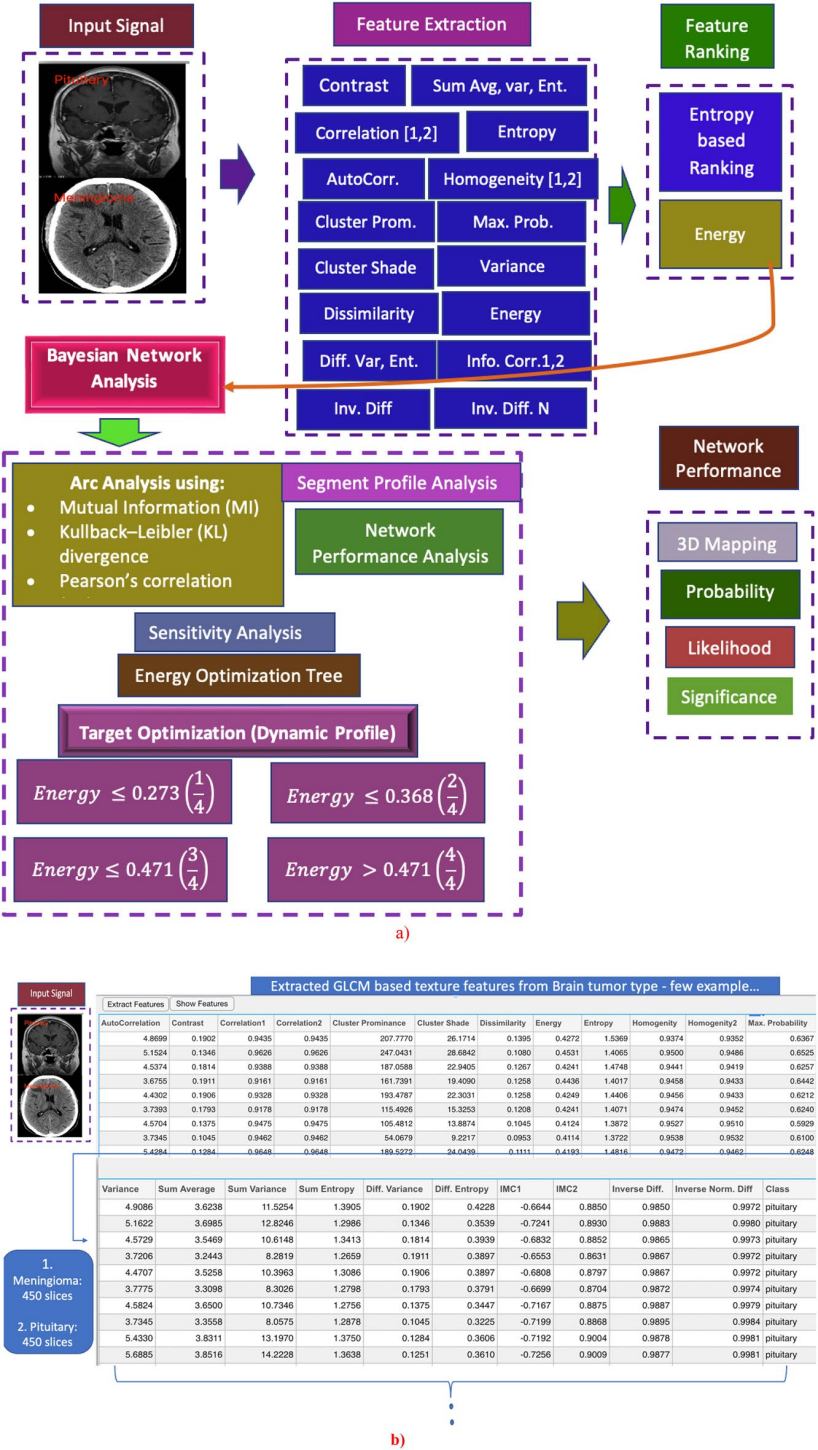
Schematic diagram based on Bayesian inference analysis on target node based on extracted GLCM based features.

The Fig. 1a shows the flow of our algorithm. We first taken brain MRI images as input and extracted the GLCM based texture feature. We then ranked the features based on entropy method. We then applied different methods of Bayesian inference by computing optimization tree, posterior probabilities, likelihood, prior and posterior means, tornado graphs, radar graphs, association of target variable with other nodes etc. The performance was evaluated, and results reveal the highly significant results. The Fig. 1b reflects the few examples of extracted GLCM features with set of quantitative values computed for each subject.

### Ethical approval

All the procedures were performed in accordance with the relevant guidelines and regulations.

## Results

In this study, we first computed the GLCM features and then ranked the features using entropy. The high ranked Energy feature was chosen as our target variable with which the further detailed analysis was done.

**Figure 2:**
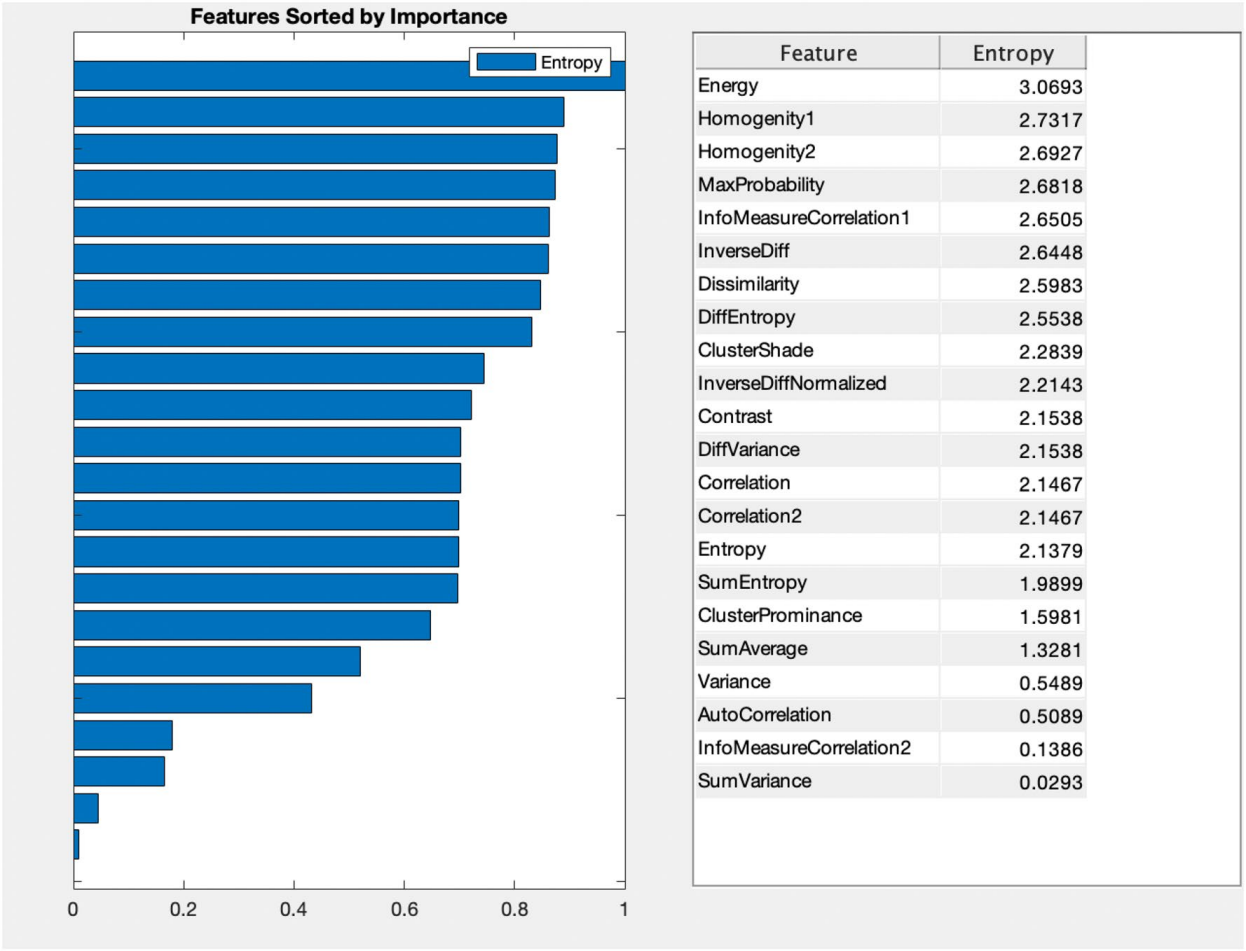
Feature ranking based on entropy values.

The Fig. 2 shows the ranking of multimodal features-based entropy values. The higher the entropy value indicates the more complex and important feature. We extracted the GLCM features from Brain tumor types. The features are ranked without utilizing any unsupervised or supervised machine learning algorithm. A specific method which ranks the features is based on the assigned score values^65^. Finally, based on these scores the features are ranked and the features with redundant information are further eliminated for classification. In this study, we ranked the GLCM features based on entropy values developed in MATLAB diagnostic tool.

**Figure 3:**
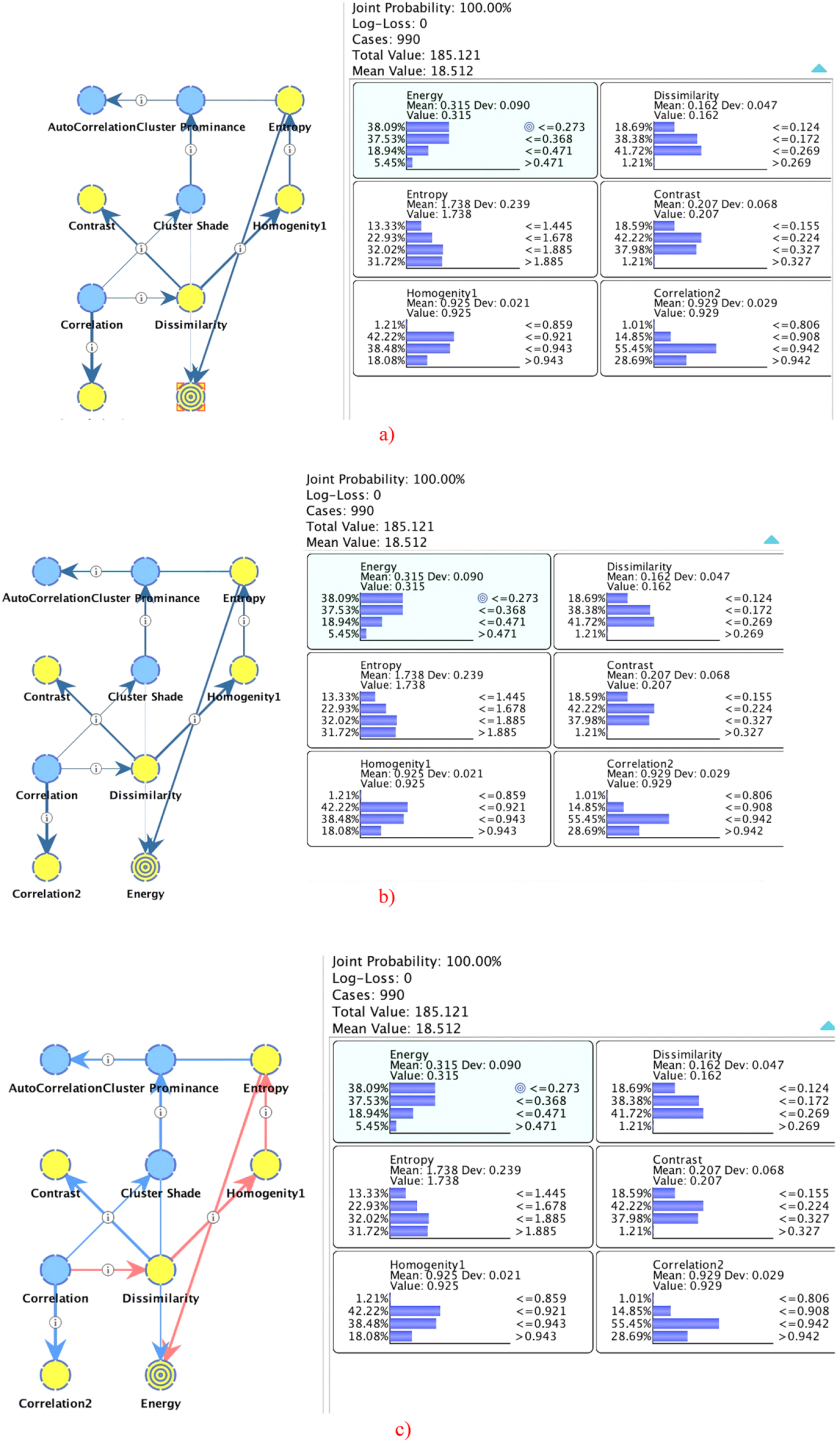
Relationship analysis using different Bayesian inference approaches such as (**a**) mutual information (MI), (**b**) Kullback–Leibler (KL) divergence, (**c**) Pearson’s correlation by applying the unsupervised learning using maximum spanning tree and selecting energy as our target node.

Figure 3 depicts the relationship analysis using Bayesian inference methods including the MI, KL and PC. The bold lines represent the stronger relationship, the lighter lines indicate the smaller relationship. The blue color indicates the positive relationship, whereas the red color indicates the negative relationship. Moreover, the arrows indicate the $(parent \to child)$ relationship. We kept our target node as Energy, using mutual information, there probability of occurrence for the state ≤ 0.273 (38.09%), state ≤ 0.368 (37.53%), state ≤ 0.471 (18.94%) and state > 0.471 (5.45%) with joint probability for all states is 100%. The probability distribution with other extracted nodes at selected states is depicted in Fig. 3a–c.

**Figure 4:**
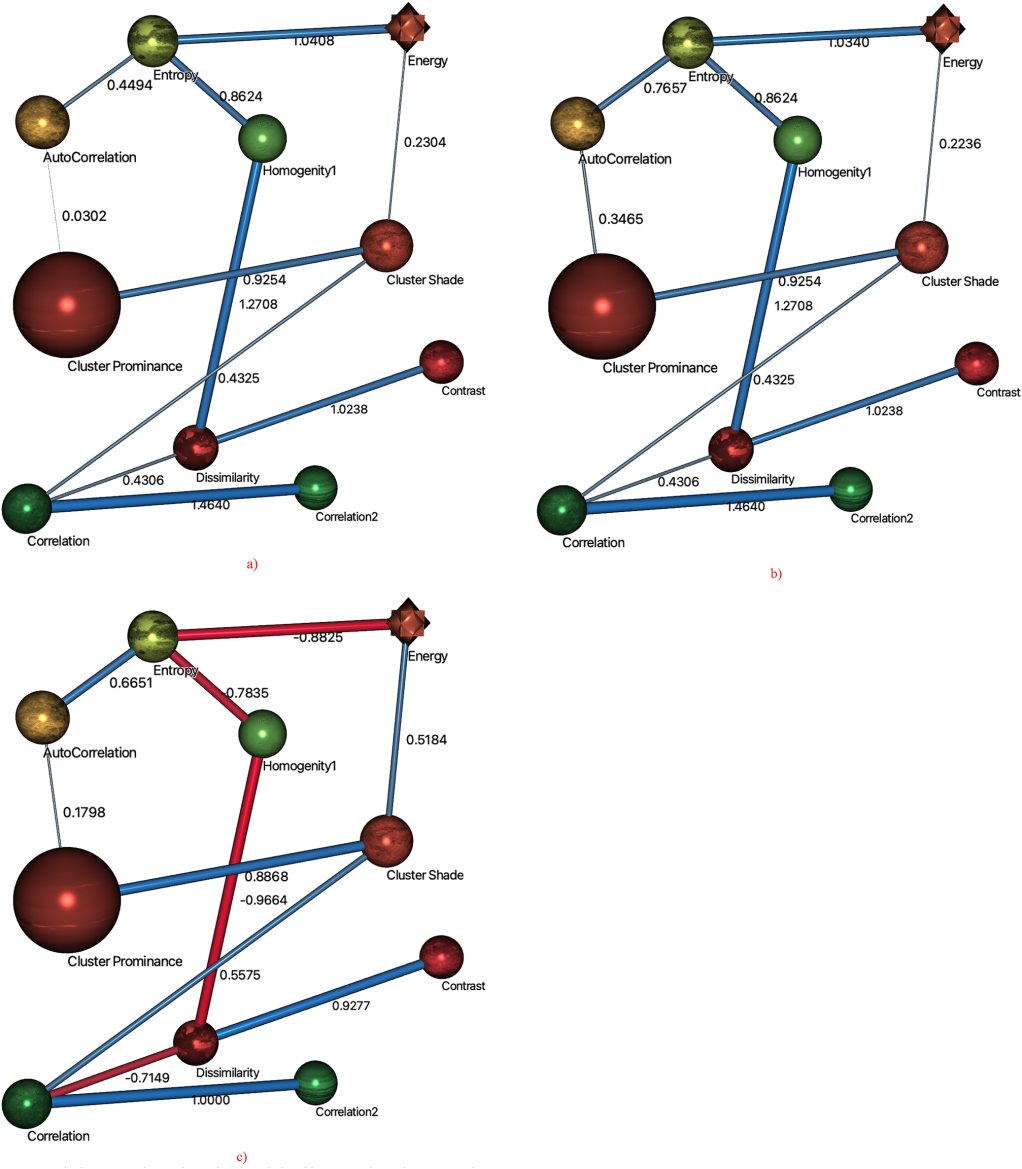
Arc analysis 3D mapping to determine the relationship among the nodes (**a**) mutual information, (**b**) Kullback–Leibler (KL) divergence, (**c**) Pearson’s correlation.

Figure 4 represents the 3D mapping arc analysis to show the relationship among the GLCM extracted features. The nodes represent the features and lines represents the relationship between the nodes. The strength of relationship is denoted by the width of line. The blue color represents the positive relationship whereas the red color denotes the negative relationship. Using the mutual information (MI), the highest strength of relationship was obtained between the nodes $Correlation \to Correlation1, \;1.4640$ followed by $Dissimilarity \to Homogenity1, \;1.2708$, $Entropy \to energy, \;1.0408$ and so on as reflected in Fig. 4a. The Fig. 4b shows the association between the nodes using KL. The highest strength of relationship was yielded between nodes $Correlation \to Correlation1, \;1.4640$ followed by $Dissimilarity \to Homogenity1, \;1.2708$, $Entropy \to energy,\; 1.0340$ and so on. The Fig. 4c denote the relationship between nodes using Pearson’s correlation. The highest strength of relationship was yielded between the nodes $Correlation \to Correlation1, \;1.000$ followed by $Dissimilarity \to homogenity1, \; - 0.9664$, $Dissimilarity \to contrast, \;0.9277$ and so on. The negative relationship was obtained between the nodes $(Correlation \to dissimilarity), \;(Dissimilarity \to homogenity1)$,$(homogenity1 \to entropy), \;(entropy \to energy)$*.* All other nodes exhibit the positive relation, where a week relationship was yielded between the nodes cluster prominence and autocorrelation. The strength of relationship using these methods is also reflected in Table 1.

**Table 1:** Parent child relationship on extracted GLCM features to distinguish the brain tumor types (pituitary and meningioma) using mutual information (MI), Kullback–Leibler (KL) divergence and Pearson’s correlation.

Parent	Child	KL divergence	Relative weight	Overall contribution (%)	Mutual information	Pearson’s correlation	p-value
Correlation	Correlation2	1.4640	1.0000	16.67	1.4640	1.0000	0.0000
Dissimilarity	Homogenity1	1.2708	0.8680	14.47	1.2708	− 0.9664	0.0000
Entropy	Energy	1.0340	0.7063	11.78	1.0408	− 0.8825	0.0000
Dissimilarity	Contrast	1.0238	0.6993	11.66	1.0238	0.9277	0.0000
Cluster shade	Cluster Prominance	0.9254	0.6321	10.54	0.9254	0.8888	0.0000
Homogenity1	Entropy	0.8624	0.5890	9.82	0.8624	− 0.78350	0.0000
Entropy	AutoCorrelation	0.7657	0.5230	8.72	0.4494	0.6651	0.0000
Correlation	Cluster Shade	0.4325	0.2954	4.92	0.4325	0.5575	0.0000
Correlation	Dissimilarity	0.4306	0.2941	4.90	0.4306	− 0.7149	0.0000
Cluster prominance	AutoCorrelation	0.3465	0.2366	3.94	0.0302	0.1798	0.0000
Cluster shade	Energy	0.2236	0.1527	2.54	0.2304	0.5184	0.0000

The Table 1 reflect the $(Parent \to child)$ relationship between the extracted GLCM features to distinguish the brain tumor types. The highest degree of relation was found between the nodes $(Correlation \to correlation2)$ yielding strength of relationship using KL and MI (1.4640), Pearson’s correlation (1.0000), with relative width 1.0000 and overall contribution of 16.67%. The contribution between other nodes was yielded such as $(Dissimilarity \to homogenity1, \;14.47\% )$, $(Entropy \to energy, \;11.78\% )$, $(Dissimilarity \to contrast, \;11.66\% )$ and so on. The highly significant results (p-value < 0.00000 was yielded for all $(Parent \to child)$ relationships.

The Table 2 reflects the incoming, outgoing and total force of different extracted GLCM features from brain tumor meningioma and pituitary. The dissimilarity node has outgoing force (2.29450), incoming force (0.4306) and total force (2.7251); the entropy node has outgoing force (1.7997), incoming (0.8624) and total force (2.6620) and so on. The highest outgoing and total force was yielded by the node dissimilarity such as 2.2945 and 2.7251 respectively. The highest incoming force was yielded by the node energy (1.2576).

**Table 2:** Node force of extracted GLCM features from brain tumor.

Node	Outgoing force	Incoming force	Total force
Dissimilarity	2.2945	0.4306	2.7251
Entropy	1.7997	0.8624	2.6620
Correlation	2.3272	0.0000	2.3272
Homogenity1	0.8624	1.2708	2.1331
Cluster shade	1.1491	0.4325	1.5816
Correlation2	0.0000	0.4640	1.4640
Cluster prominance	0.3465	0.9254	1.2719
Energy	0.0000	1.2576	1.2576
AutoCorrelation	0.0000	1.1121	1.1121
Contrast	0.0000	1.0238	1.0238

**Figure 5:**
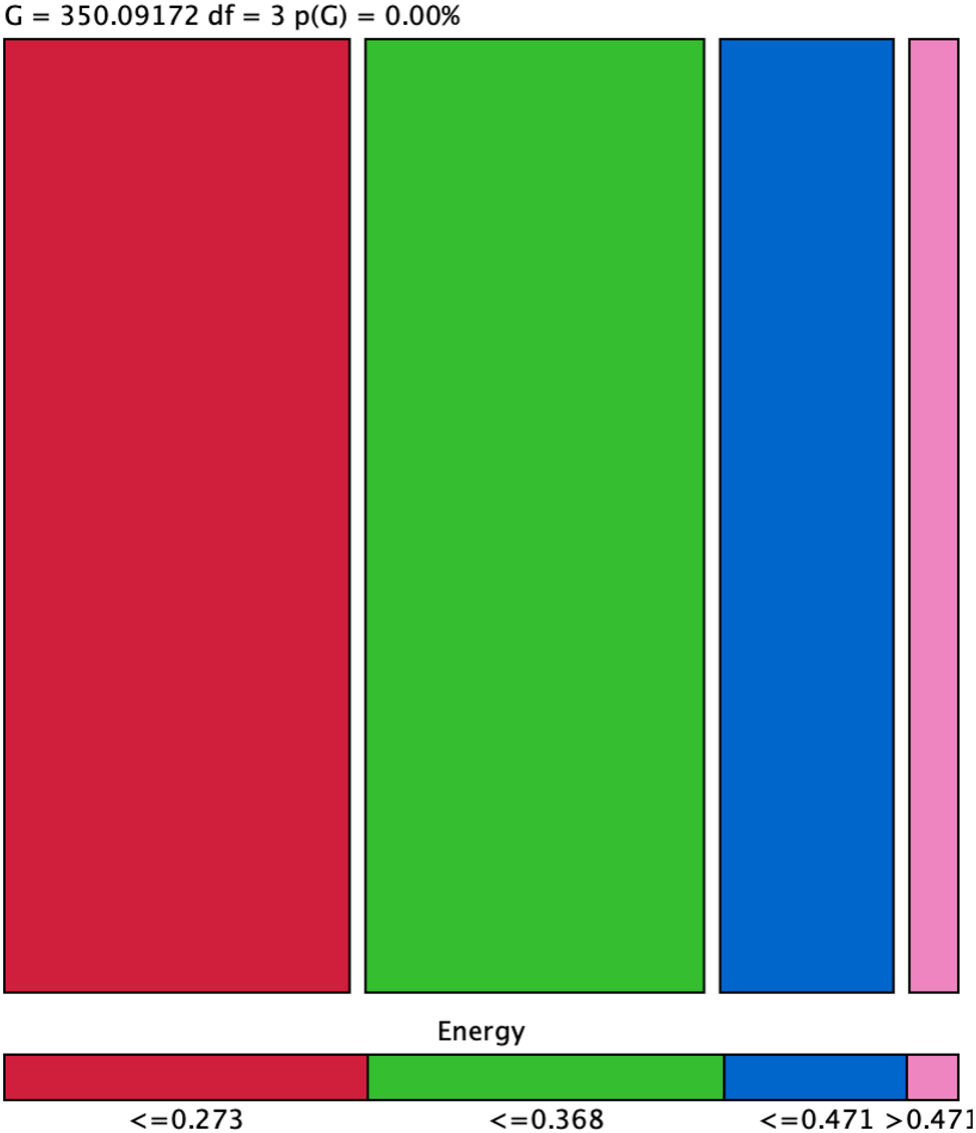
Analysis of target node energy with other extracted nodes using mosaic graph based on selected target node energy and predictions of occurrence made against each state out of total (i.e. ≤ 0.273, 354/394; ≤ 0.368, 321/374; ≤ 0.471, 143/192; > 0.471, 27/28).

We randomly chosen the subjects i.e. Pituitary (495 images) and Meningioma (495 images) with a total of 990 images. We ranked the features before applying the Bayesian inference approach. The Energy was highly ranked features measured using EROC and random classifier slope, which was selected as our target for further Bayesian analysis. We computed the association of top ranked Energy feature with other features to further unfold the association among the features. There were four states represented by ≤ 0.273 (394 images), ≤ 0.368 (374 images), ≤ 0.471 (191 images) and > 0.471 (31 images) as represented in the Mosaic association graph in Fig. 5 and Table 3. After prediction, 354 were predicted for first class, 321 for second class, 145 for third class and 30 for fourth class. The reliability and precision for class 1 to 4 was yielded according as 89.84%, 85.82%, 75.92%, 96.77% and 94.65%, 85.37%, 81.00%, 49.18% respectively.

**Table 3:** Analysis of target node energy with other extracted nodes.

State	Purity (%)	Neighborhood
> 0.471 (4/4)	96.77	≤ 0.471 (3/4)
≤ 0.273 (1/4)	89.84	≤ 0.471 (3/4)
		≤ 0.273 (1/4)
≤ 0.368 (2/4)	85.82	≤ 0.471 (3/4)
		≤ 0.273 (1/4)
≤ 0.471 (3/4)	75.92	> 0.471 (4/4)
		≤ 0.368 (2/4)

The Table 4 reflects the overall analysis of target node energy with other nodes. All nodes exhibits the highly significant results.

**Table 4:** Overall analysis of target node energy with other extracted GLCM features.

Node	Mutual information (MI)	Normalized MI (%)	Relative significance	Prior mean	p-value
Entropy	1.0408	52.10	1.0000	1.7381	0.00000
Homogenity1	0.6500	32.50	0.6245	0.9252	0.00000
Dissimilarity	0.5835	29.17	0.5606	0.1625	0.00000
Contrast	0.4336	21.68	0.4166	0.2066	0.00000
Correlation	0.2955	14.78	0.2840	0.9287	0.00000
Correlaltion2	0.2955	14.78	0.2840	0.9287	0.00000
AutoCorrelation	0.2410	12.04	0.2315	5.5282	0.00000
Cluster shade	0.2304	11.52	0.2214	17.70	0.00000
Cluster prominance	0.1675	8.37	0.1609	156.68	0.00000

**Figure 6:**
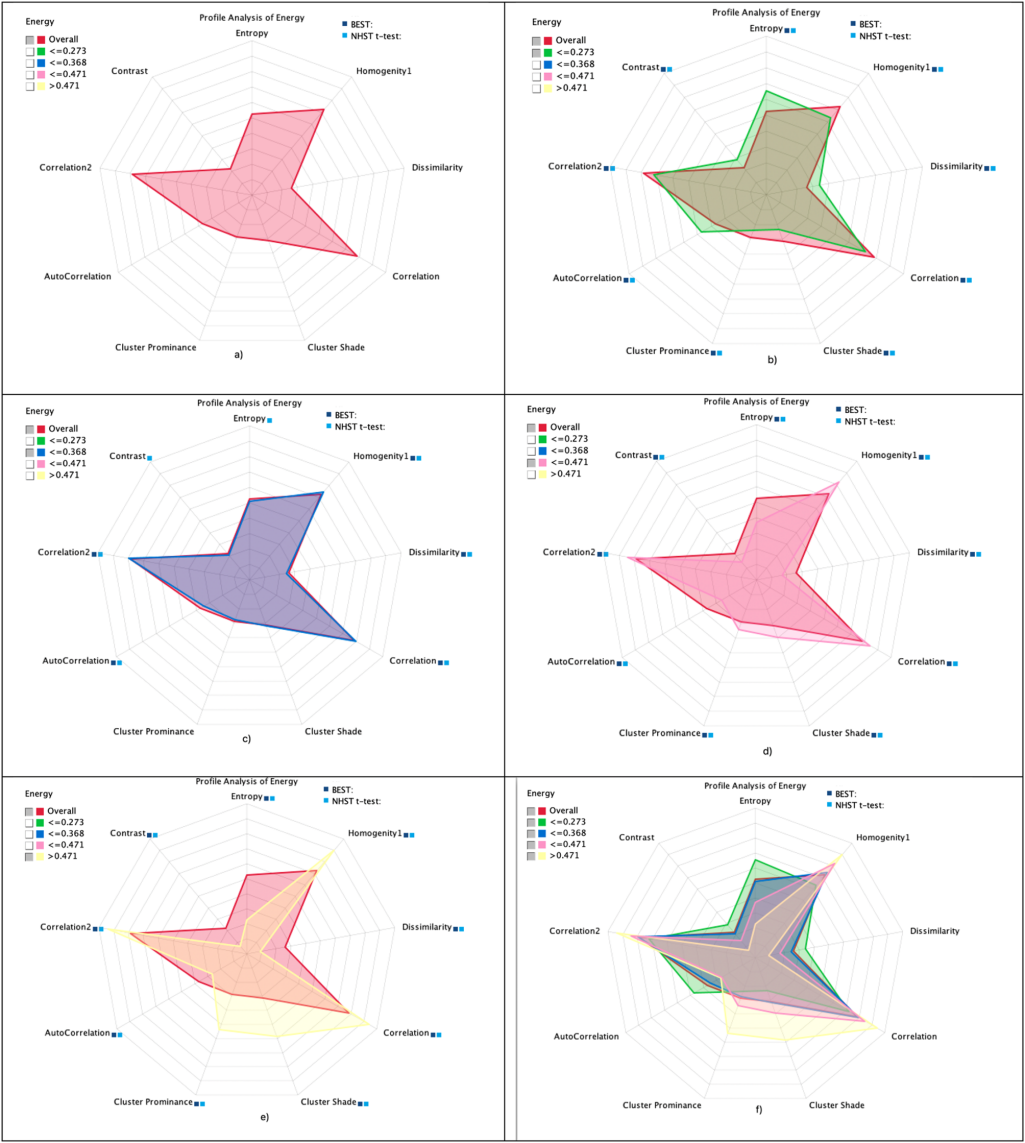
Association graph of segment profile analysis of energy node with other extracted GLCM features using radar chart graph at different selected states (**a**) overall and overall with selected states (**b**) ≤ 0.273, (**c**) ≤ 0.368, (**d**) ≤ 0.471, (**e**) > 0.471, overall with (**f**) all selected states.

The Fig. 6 depicts the analysis of association graph for segment profile analysis of top ranked target node with other extracted GLCM features using the Radar chart which reflect the distributions based on 1 to 12 clock hours. The Fig. 6a reflect the overall probability and we used the NHST *t* test and Bayesian test to find the significance to distinguish with other different states such as (b) ≤ 0.273, (c) ≤ 0.368, (d) ≤ 0.471, (e) > 0.471 as reflected in Fig. 6b–e. The clusters ≤ 0.273, ≤ 0.471 and > 0.471 using both the test yielded the highly significant results with all the extracted GLCM features. The state ≤ 0.368 yielded high significant results using both test with homogenity1, dissimilarity, correlation, correlation2 and autocorrelation, whereas significant results using NHST *t* test with contrast and energy, while no significant results were yielded with cluster shade and cluster Prominance.

**Figure 7:**
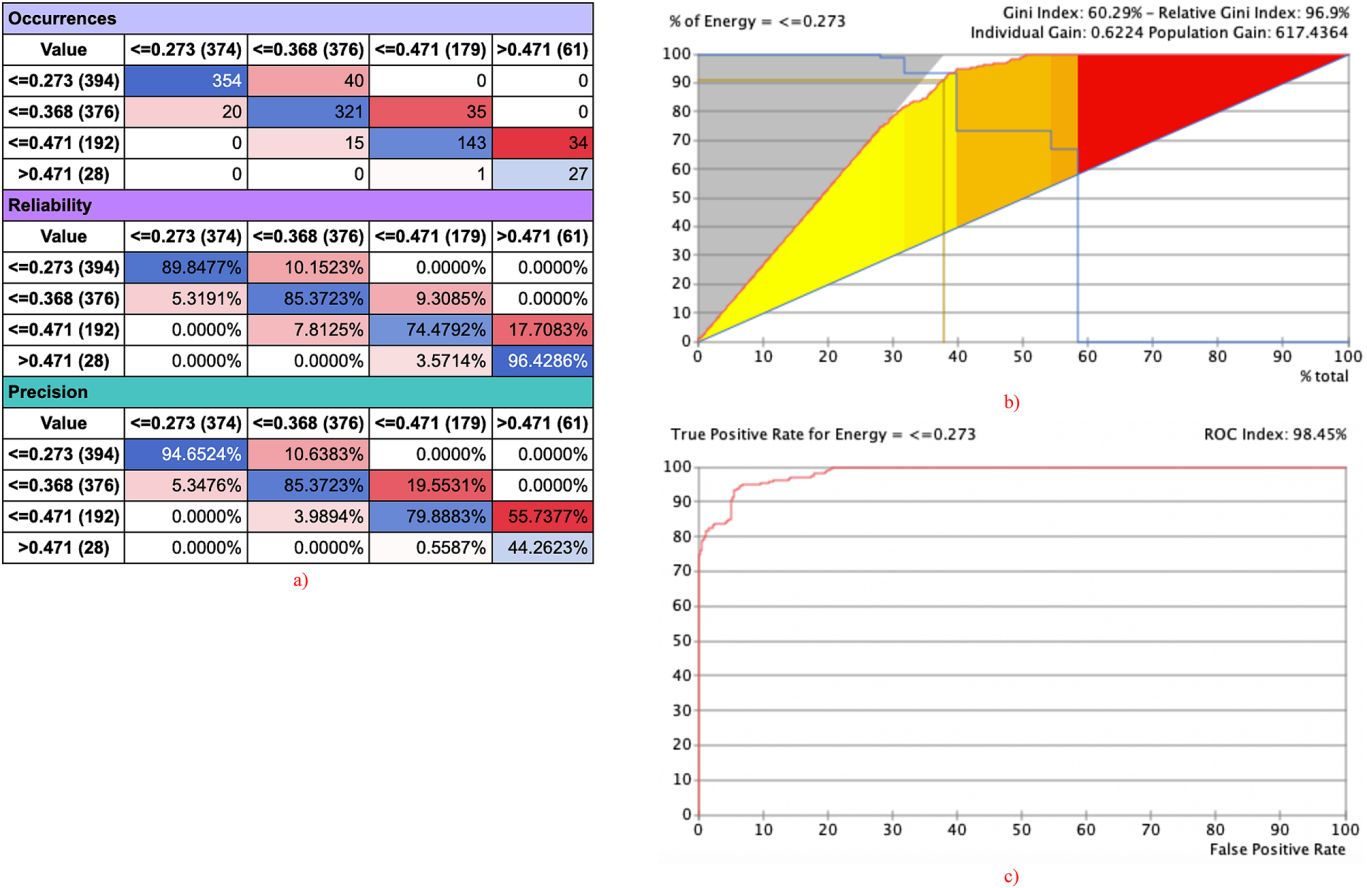
Network performance target evaluation energy node with other selected nodes (**a**) occurrence, reliability and precision report; (**b**) gain report of state ≤ 0.273, (**c**) ROC index of state ≤ 0.273.

The network performance of selected target node Energy with other selected nodes yielded R of 0.9497, R2 of 0.9019, RMSE of 0.0290 and NRMSE of 0.0490. The selected state ≤ 0.273 yielded the highest predictions with 89.84% of reliability, 96.65% if precision and 98.49% of ROC index as depicted in Fig. 7a–c.

**Figure 8:**
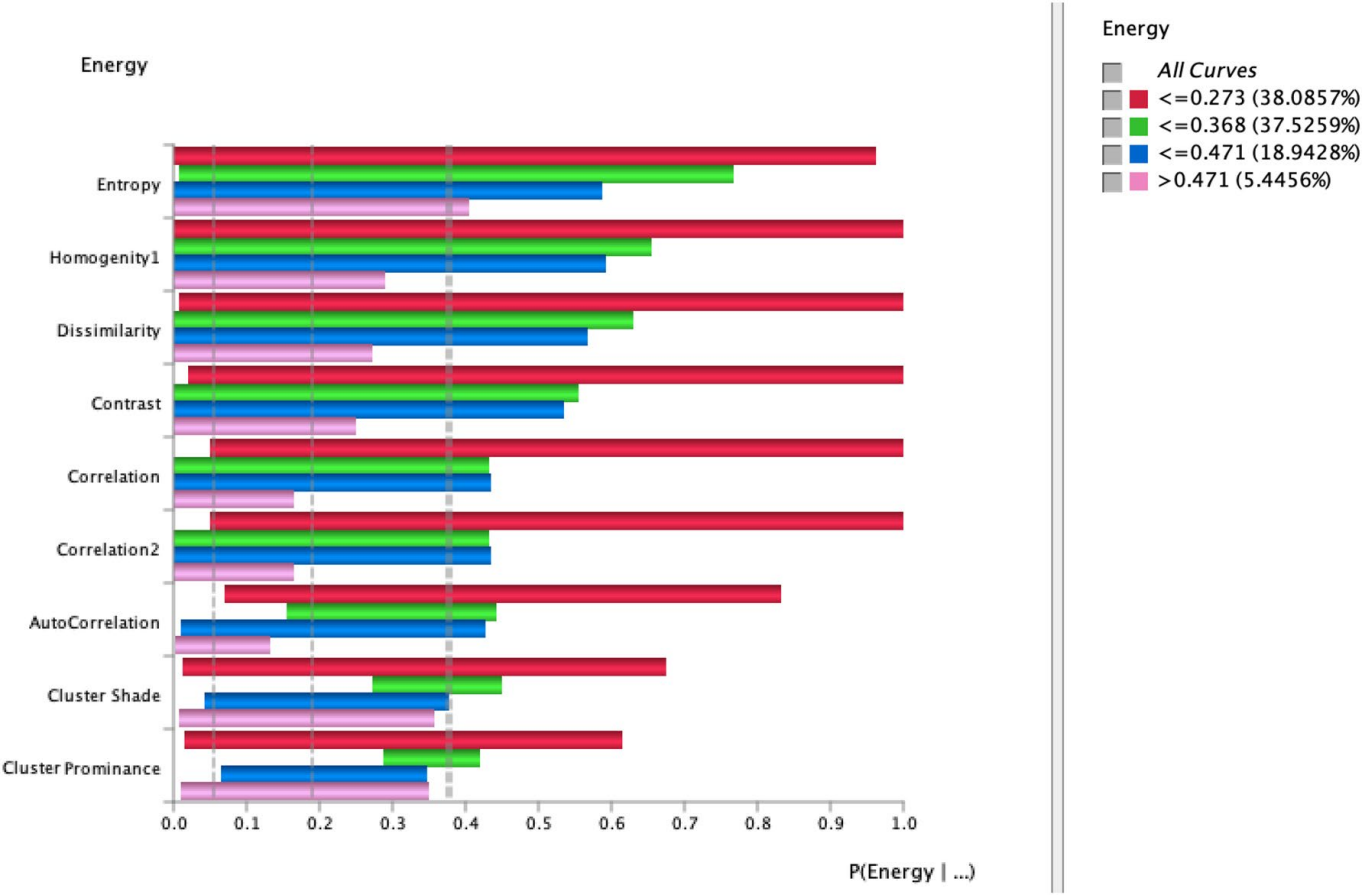
Tornado diagram of posterior probabilities to compute the significance of Energy node with all nodes at selected cluster states (≤ 0.273, ≤ 0.368, ≤ 0.471, and > 0.471).

Using the tornado graph as reflected in Fig. 8, we visualize the maximum deltas in the posterior probabilities of the target states and hard evidence is set on the selected variables. The strong deltas are shown at the top of the graph. The highest association was yielded with entropy, homogeneity, dissimilarity, contrast, correlation, correlation2 cluster state ≤ 0.273 followed by cluster state ≤ 0.368, ≤ 0.471 and > 0.471 reflected in Fig. 8. This indicates that high top ranked Energy feature prevails high associations with entropy, homogeneity, dissimilarity, contrast, correlation, correlation2 which can be used as better predictor for improved diagnosis and prognosis of brain tumor types. The association of highest ranked Energy node with other nodes in the state ≤ 0.368 was obtained with entropy, homogeneity, dissimilarity.

**Figure 9:**
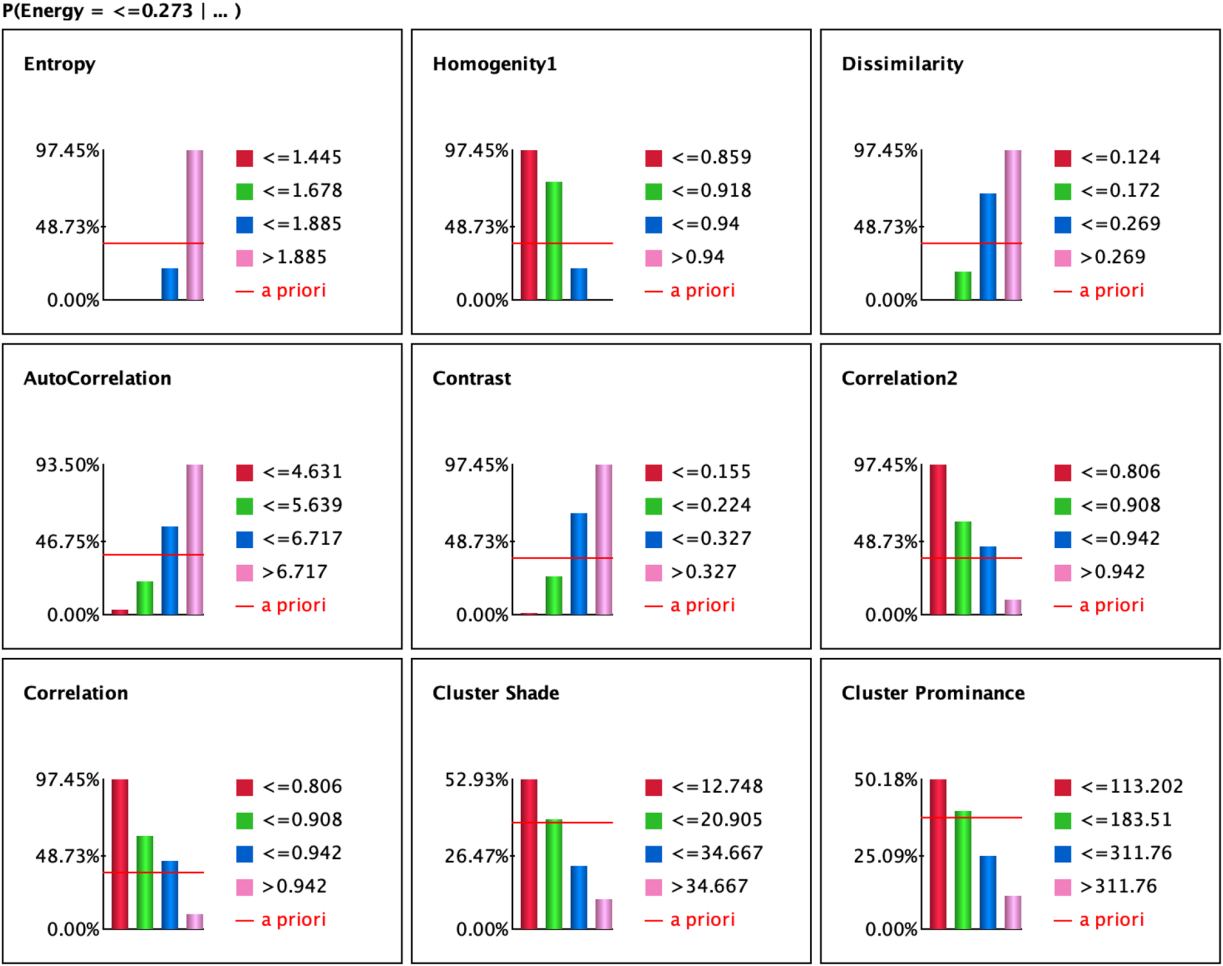
Target’s posterior probabilities for outcome variable Energy = ≤ 0.273.

The Fig. 9 denote the target’s posterior probabilities for the selected target variable Energy at state ≤ 0.273. The prior value is denoted by red line. The bar exceeding the red line indicates that variables values influencing the target variable.

**Figure 10:**
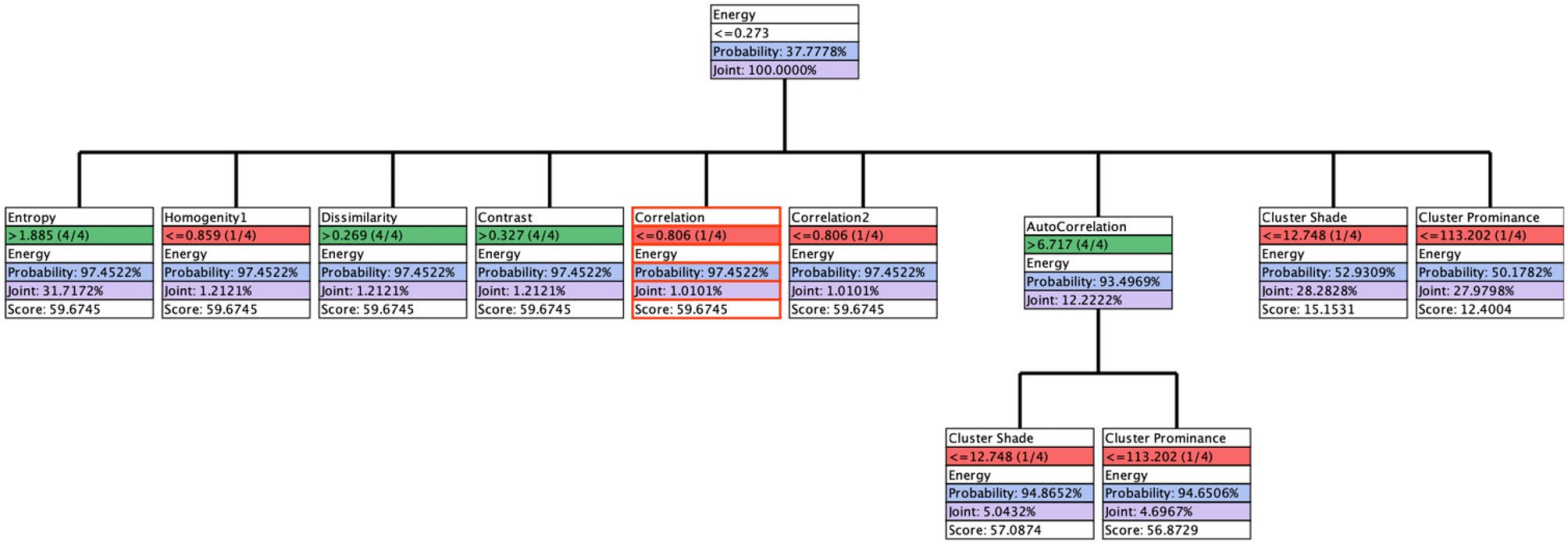
Tree optimization of GLCM energy as target node.

Our optimization target state is ≤ 0.273. The Fig. 10 indicates that we have multiple pathways to get into the Energy with a 94% or higher probability. The Table 6 reflect the target node Energy at cluster state ≤ 0.273. With the Entropy node at 1.885, a highest posterior probability was obtained P (s|H) of 97.45%, Likelihood P(H|s) of 81.81%, Bayes factor of 2.57% and generalized Bayes factor of 9.68%. The prior values and posterior values of other nodes are reflected in this Table 6.

The Table 5 summarize the dynamic profile of all the clusters. The dynamic profile uses the greedy search algorithm to simulate set of evidence for maximizing the probability of selected clusters.

**Table 5:** Search method: hard evidence dynamic profile energy: probability maximization (likelihood) energy ≤ 0.273 (1/4).

Node	Hypothesis	Posterior probability P (s|H) (%)	Marginal likelihood P(H) (%)	Likelihood P(H|s) (%)	Bayes factor BF (s, H) (%)	Generalized BF GBF (s, H)
A priori		37.77	100			
Entropy	> 1.885 (4/4)	97.45	31.71	81.81	2.57	9.68

Node	Prior value/mean	Posterior value/mean				
Homogeneity	0.9252	0.9075				
Dissimilarity	0.1625	0.1992				
Correlation	0.9287	0.9137				
Correlation2	0.9287	0.9137				
Cluster shade	17.70	14.72				
Cluster prominence	156.67	134.09				
Contrast	0.2066	0.25296				
Autocorrelation	5.52	6.4093				

The Table 6 reflect the target node Energy at cluster state ≤ 0.368. With the Entropy node at 1.885, a posterior probability was obtained P (s|H) of 78.86%, marginal likelihood (32.02%), Likelihood P(H|s) of 66.48%, Bayes factor of 2.07% and generalized Bayes factor of 4.21%. as also reflected in Fig. 6. The prior values and posterior values of other nodes are reflected in this Table 7.

**Table 6:** Search method: hard evidence dynamic profile energy: probability maximization (likelihood) energy ≤ 0.368 (2/4).

Node	Hypothesis	Posterior probability P (s|H) (%)	Marginal likelihood P(H) (%)	Likelihood P(H|s) (%)	Bayes factor BF (s, H) (%)	Generalized BF GBF (s, H) (%)
A priori		37.97	100			
Entropy	> 1.885 (3/4)	78.86	32.02	66.48	2.07	4.21

Node	Prior value/mean	Posterior value/mean				
Homogeneity1	0.9252	0.9075				
Dissimilarity	0.1625	0.1672				
Correlation	0.9287	0.9282				
Correlation2	0.9287	0.9282				
Cluster shade	17.70	17.11				
Cluster Prominence	156.67	151.90				
Contrast	0.2066	0.2131				
Autocorrelation	5.52	5.52

**Table 7:** Search method: hard evidence dynamic profile energy: probability maximization (likelihood) Energy ≤ 0.471 (3/4).

Node	Hypothesis	Posterior Probability P (s|H) (%)	Marginal Likelihood P(H) (%)	Likelihood P(H|s) (%)	Bayes Factor BF (s, H) (%)	Generalized BF GBF (s, H) (%)
A Priori		18.08	100			
Entropy	≤ 1.445 (1/4)	53.03	13.33	39.10	2.93	4.17

Node	Prior value/mean	Posterior value/mean				
Homogeneity1	0.9252	0.9075				
Dissimilarity	0.1625	0.1090				
Correlation	0.9287	0.9477				
Correlation2	0.9287	0.9477				
Cluster shade	17.70	22.44				
Cluster Prominence	156.67	193.05				
Contrast	0.2066	0.1367				
Autocorrelation	5.52	4.59				

The Table 7 reflect the target node Energy at cluster state ≤ 0.471 With the Entropy node at ≤ 1.445, a posterior probability was obtained P (s|H) of 53.03%, marginal likelihood (13.33%), Likelihood P(H|s) of 39.10%, Bayes factor of 2.93% and generalized Bayes factor of 4.17%. as also reflected in Fig. 6. The prior values and posterior values of other nodes are reflected in this Table 8.

**Table 8:** Search method: hard evidence dynamic profile energy: probability maximization (likelihood) energy > 0.471 (4/4).

Node	Hypothesis	Posterior Probability P (s|H) (%)	Marginal Likelihood P(H) (%)	Likelihood P(H|s) (%)	Bayes Factor BF (s, H) (%)	Generalized BF GBF (s, H)
A Priori		6.16	100			
Entropy	≤ 1.445 (1/4)	46.21	13.33	100	7.50%	58,546,795,816,448

Node	Prior value/mean	Posterior value/mean				
Homogeneity1	0.9252	0.9507				
Dissimilarity	0.1625	0.1090				
Correlation	0.9287	0.9477				
Correlation2	0.9287	0.9477				
Cluster shade	17.70	22.44				
Cluster prominence	156.67	193.05				
Contrast	0.2066	0.1367				
Autocorrelation	5.52	4.59				

The Table 8 reflect the target node Energy at cluster state > 0.471 with the Entropy node at ≤ 1.445, a posterior probability was obtained P (s|H) of 46.21%, marginal likelihood (13.33%), Likelihood P(H|s) of 100%, Bayes factor of 7.50%. The prior values and posterior values of other nodes are reflected in this Table 4.

## Discussion

The Bayesian networks (BNs) are combination of probability theory and graph, which are capable to capture efficiently the most significant causation factor in the pathological subjects and can capture the relationship between different causal relationship^82^. BNs effectively assess the cause-consequence analysis from extracted GLCM features of Brain MRIs^87^. The detailed Bayesian analysis utilizing relationship analysis, segment profile analysis using radar chart, tornado diagrams of posterior probabilities, and network performance analysis can successfully be utilized for treatment planning and improved diagnosis of target node with other extracted nodes. These networks provide an efficient tool for detailed analysis to determine the interconnectivity and association between the variable of interest^88^. The BNs comprised of qualitative and quantitative analysis. The qualitative analysis depicts the structure of the graph by expressing the graphical representation in terms of cause relationship of variable of interest^89^. The quantitative portion of the graph quantify the associations with conditional probabilities among the variables and target state according to cause order or connectivity. BNs, apart from not only determine the causal relationship, but also compute the nature of relationship between the factors involved^90^. Moreover, these networks are also more robust and capable to determine the genuine graphical and visual relationship between variables involved. BNs are capable to process the data and ambiguity of all states of a variable using inference in a probabilistic system. These networks are also suitable for decision-making processes by providing consistent, scrupulous, and systemized assessment. For our extracted GLCM features, we first ranked the features based on entropy value. The top ranked energy feature was set as our target node, and we further conducted the sensitivity analysis, segment profile analysis, and network analysis with our target node. The BayesiaLab through tornado chart identify those variables which are most critical from the perspective of their effect on the target variable and provide the contributions of their probabilities of respective variables. The variable with maximum and prominent sensitivity are presented in tornado graph. The cluster state ≤ 0.273 yielded the highest association for variables homogeneity, entropy, dissimilarity, correlation, contrast and correlation2.

The BN is a pictorial illustration by computing the joint probability distribution. The Bayesian network structure comprised on nodes which denotes the random variables, and arcs reflect the dependence structure reflecting causality between the variables. When there is absence of arc between the nodes, it denotes that variable are conditionally independent. Bayesian network structure is either supervised or unsupervised, however, joint probability distribution is unsupervised. Bayesian network have been utilized to analyse the uncertainties and covariations among the multiple variables^91^. We used the learning based on unsupervised learning using maximum spanning tree by setting mining description length as learning setting and taboo list size of 45. The inference was made based on the adaptive questionnaire by setting Energy highly ranked feature as our target node and computed its association among other variables. Presently, the researchers are devolving tools using machine learning methods. For machine learning, the most important step is to compute the most relevant features. However, extracting the most relevant features is still a challenging task as all the extracted features are not equally important. We, therefore, first ranked the extract GLCM based texture features. The highest ranked feature was energy (3.0693) followed by homogenity1 (2.7317), homogenity2 (2.6927), max. probability (2.6818) and so on. The least significant feature was sum variance (0.0293). This indicates that the energy feature contribute more than other extracted GLCM features to distinguish the pituitary from meningioma. We first applied the unsupervised maximum spanning tree algorithm and kept energy as our target variable to determine the further associations with other features to unfold hidden dynamics with the top ranked feature. The correlation, strength of relationship and degree of relationship among the $(Parent \to child)$ node was computed using the mutual information, Kullback–Leibler (KL) divergence and Pearson’s correlation. We then computed the incoming, outgoing and total force between the nodes to further determine the comprehensive relationship between the nodes. The analysis was also done using tornado diagram, network performance, and segment profile analysis using the radar chart. The tornado graph indicates that the selected high ranked target variable Energy has highly significant results with most of the nodes at the selected cluster states i.e. ≤ 0.273, ≤ 0.368, ≤ 0.471 and > 0.471. Moreover, high occurrences, and reliable results were yielded at the selected states to distinguish the pituitary from meningioma. The tornado diagram also indicates that higher associations of Energy variable at selected cluster state ≤ 0.273 were yielded with variable entropy, homogeneity, dissimilarity, contrast, correlation, correlation2. The target’s posterior probabilities also indicates that the selected target Energy node shows the high influence with other nodes. A high ROC index and Gini index were yielded to distinguish these states.

The researchers in the past utilized different imaging analysis methods for diagnosing the MRI images^92–94^. Hussain et al.^38^ applied Bayesian inference approach to compute the association among the morphological features extracted from Prostate cancer. Most of these studies were relied on classification tasks. The author obtained the classification performance with accuracy 91.28%^44^, 84.19%^95^, 86.56%^96^, 90.89%^97^, 84.19%^98^, and 99%^18^. Previous studies relies on classification methods. However, this novel technique is proposed to further investigate the dynamics, associations, posterior probabilities, prior probabilities, marginal likelihood, prior means and posterior means to further unfold the relevance and relationships among the extracted features. The proposed approach will be very helpful for improved diagnosis and prognosis of brain tumor types.

## Conclusions

In this study, we first computed the GLCM features from brain tumor subtypes i.e., pituitary and meningioma MRIs. We then ranked the features based on the entropy ranking method. The high ranked energy feature was used as our target variable. We then applied the Bayesian approach to further compute the association, arc analysis, tree optimization, dynamic profiling. The proposed methods further unfold the dynamics which can be helpful to understand the association, dynamic profiling of computed features for better diagnostic system of brain tumor types. The Bayesian inference approach can be used as a new biomarker to comprehend a detailed analysis of extracted variables to further unfold the underlying dynamics present the computed future for further improved prognosis, diagnosis and treatment planning to achieve better clinical outcomes. In future, we will further extend more Bayesian inference methods and other tumor types with clinical details and larger dataset.

## Data Availability

The use of all data mentioned in this article is publicly available^43,44^ (https://github.com/chengjun583/brainTumorRetrieval).
